# Circular RNA circ‐CMPK1 contributes to cell proliferation of non‐small cell lung cancer by elevating *cyclin D1* via sponging miR‐302e

**DOI:** 10.1002/mgg3.999

**Published:** 2019-12-21

**Authors:** Dong Cui, Runlin Qian, Yin Li

**Affiliations:** ^1^ Department of Thoracic Surgery The Affiliated Cancer Hospital of Zhengzhou University Zhengzhou P.R. China; ^2^ Department of Thoracic Surgery Henan Chest Hospital Zhengzhou P.R. China

**Keywords:** biomarker, ceRNA, circular RNA, microRNA, NSCLC, proliferation

## Abstract

**Background:**

It is well recognized that competing endogenous RNA (ceRNA) regulatory network is linked to the development and progression of cancer, including non‐small cell lung cancer (NSCLC). Herein, we aimed to explore the functional role of circ‐CMPK1/miR‐302e/*cyclin D1* ceRNA signaling in NSCLC.

**Methods:**

GEO database (http://www.ncbi.nlm.nih.gov/geo/query/acc.cgi?acc=GSE102287) was utilized to screen differentially expressed miRNAs in NSCLC. Quantitative reverse transcription PCR (qRT‐PCR) and western blotting assays were used to determine gene expression. Cell proliferation analysis was performed with Cell Counting Kit‐8 (CCK‐8) and cell cycle assays. Luciferase reporter and RNA pull‐down assays were conducted to identify the interaction among circ‐CMPK1, miR‐302e, and *cyclin D1*. Xenograft tumor model was established to evaluate the role of circ‐CMPK1/miR‐302e/*cyclin D1* axis in vivo.

**Results:**

miR‐302e expression was significantly down‐regulated in NSCLC cell lines and tissues and its decrease was closely associated with aggressive clinicopathological features and unfavorable outcome. Overexpression and knockdown of miR‐302e obviously retarded and enhanced the growth of NSCLC, respectively. Furthermore, we found that miR‐302 was sponged by circular RNA *CMPK1* (circ‐CMPK1, hsa_circ_0012384), which was remarkably up‐regulated in NSCLC and predicted poor prognosis. Circ‐CMPK1 was capable to promote NSCLC cells proliferation by increasing the expression of *cyclin D1* via inhibiting miR‐302 activity. Moreover the miR‐302e‐mediated tumor inhibition could be effectively counteracted by ectopic expression of circ‐CMPK1 or *cyclin D1* both in vitro and in vivo.

**Conclusion:**

Our data demonstrate for the first time that circ‐CMPK1/miR‐302e/*cyclin D1* signaling plays an essential regulatory role in NSCLC and targeting this axis may be an efficacious avenue for treatment of NSCLC patients.

## Graphical abstract

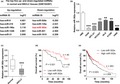

## 1 INTRODUCTION

Non‐small cell lung cancer (NSCLC), accounting for approximately 80%–85% of all lung cancer subtypes, is the most common cause of cancer‐related deaths worldwide (Herbst, Morgensztern, & Boshoff, 2018). Although the early detection and treatment of NSCLC have been greatly improved in recent decades, the 5‐year survival rate of NSCLC patients still remains very low, mainly due to more than 70% of patients present with the middle or advanced stage at the time of diagnosis (Osmani, Askin, Gabrielson, & Li, 2018). Therefore, in‐depth analysis of the pathogenesis of NSCLC will provide a theoretical basis for improving the diagnosis and treatment for patients with NSCLC.

microRNA (miRNA) is a small endogenous non‐coding RNA molecule consisting of approximately 21–25 nucleotides (Chipman & Pasquinelli, 2019). It is transcribed into a primary miRNA (pri‐miRNA) in the nucleus, and then processed by the RNase III nuclease Drosha/pasha into a hairpin‐like precursor miRNA (pre‐miRNA) of about 70–90 nucleotides, which is finally transported to the cytoplasm and processed into mature miRNA by Dicer (Bracken, Scott, & Goodall, 2016). Multiple lines of evidence suggests that miRNA controls gene expression and cell function by promoting the degradation or inhibiting translation of its downstream target genes via pairing with the 3′‐UTR or CDS sequence of target genes (Zhang, Cozen, Liu, Chen, & Lowe, 2016). It is well accepted that miRNA participates in the occurrence and progression of cancer including NSCLC, such as miR‐3607‐3p (Gao, et al., 2018), miR‐19 (Peng, Guan, & Gao, 2018), miR‐483‐3p (Yue et al., 2018), miR‐647 (Zhang, et al., 2018), miR‐455‐3p (Gao, et al., 2018), miR‐205 (Park et al., 2014), etc., which act as an oncogene or tumor suppressor gene by altering different signaling pathways in NSCLC. Nevertheless, the biological role of miR‐302e in NSCLC remains unknown.

Circular RNA (circRNA), a special type of non‐coding RNA with covalently closed loop, is abundantly expressed in eukaryotes and has been found to harbor important gene‐regulatory potential in recent years (Kristensen, Hansen, Veno, & Kjems, 2018; Rybak‐Wolf et al., 2015). Emerging evidence shows that circRNA is linked to tumorigenesis and aggressiveness by functioning as a competing endogenous RNA (ceRNA) via sponging miRNAs (Hansen et al., 2013; Zhong et al., 2018). For instance, circ‐UBAP2 (Wang et al., 2018), circ‐HIPK3 (Zeng, et al., 2018), and circ‐SMAD2 (Zhang, et al., 2018), could sponge miR‐661, miR‐7, and miR‐629 to regulate the progression of triple‐negative breast cancer, colorectal cancer, and hepatocellular carcinoma, respectively. However, the ceRNA regulatory role of circRNA in NSCLC is still poorly characterized.

In this study, we investigated the functional and clinical implications of miR‐302e in NSCLC and further elucidated the exact role of the circ‐CMPK1/miR‐302e/*cyclin D1* (Gene ID: 595) regulatory network in the progression of NSCLC.

## 2 MATERIALS AND METHODS

### 2.1 Ethical compliance

This study was approved by the Ethics Committee of HeNan Provincial Chest Hospital (NO. HNPCH1523).

### 2.2 Clinical tissue samples

A total of 80 pairs of NSCLC and adjacent normal tissue samples was obtained from patients diagnosed with NSCLC who underwent surgical resection in HeNan Provincial Chest Hospital. None of the patients received anti‐tumor treatment before surgery. All specimens were immediately frozen and stored in liquid nitrogen after resection. The detail clinicopathological parameters of patients are described in Table 1. The written informed consent of all patients was also achieved.

**Table 1:** Correlation between miR‐302e expression and clinicopathological characteristics in 80 NSCLC patients

Parameters	All cases	miR‐302e expression		*p* value
		Low	High	
Gender				
Male	56	27	29	.626
Female	24	13	11	
Age (years)				
≤60	37	17	20	.501
>60	43	23	20	
Smoking status				
Smokers	45	21	24	.499
Non‐smokers	35	19	16	
Tumor size				
≤3	29	9	20	**.011**
>3	51	31	20	
Lymph node metastasis				
No	48	19	29	**.022**
Yes	32	21	11	
TNM stage				
I‐II	53	21	32	**.009**
III‐IV	27	19	8	
Differentiation				
Well/moderate	52	21	31	**.019**
Poor	28	19	9	

Blod values indicates *p* < .05.

### 2.3 Cell culture and transfection

All NSCLC cell lines including A549, SPC‐A1, HCC827, 95‐D, H1299, H460 and a normal human bronchial epithelial (HBE) cell were purchased from the Chinese Academy of Science and routinely grown in DMEM or RPMI‐1640 medium with 10% fetal bovine serum. Cell transfection was carried out by using Lipofectamine 2000 (Invitrogen) based on the manufacturer's instructions. miR‐302e mimics and inhibitors were obtained from Gene‐Pharma company, circ‐CMPK1 and *cyclin D1* overexpression vectors were purchased from Geneseed and Applied Biological Materials companies, respectively.

### 2.4 Quantitative reverse transcription PCR (qRT‐PCR)

Total RNA in NSCLC cell lines and tissues was extracted with TRIzol reagent (Invitrogen) according to manufacturer's protocols and then synthesized into single‐stranded cDNA. Next, quantitative PCR was conducted by using SYBR Green SuperMix (Roche) with specific primers under the following cycling conditions (10μl total volume with 40 cycles): 95°C for 10s, 56°C for 20s, 72°C for 30s. *U6* and *β‐actin* were used as endogenous references of miR‐302e and circ‐CMPK1/*cyclin D1*, respectively. 2^−ΔΔCt^ calculation method was employed to assess relative RNA expression. The specific primer sequences are as follows:

circ‐CMPK1: Forward: 5′‐TCCATTCAGTGATGTTGGATG‐3′,

Reverse: 5′‐TTCAAGGATGCCTTCAGGTT‐3′;

*cyclin D1*: Forward: 5′‐AACTACCTGGACCGCTTCCT‐3′,

Reverse: 5′‐TCGGTGTAGATGCACAGCTT‐3′;

*β‐actin*: Forward: 5′‐CGTACCACTGGCATCGTGAT‐3′,

Reverse: 5′‐GTGTTGGCGTACAGGTCTTTG‐3′;

### 2.5 Cell Counting Kit‐8 (CCK‐8) and cell cycle assays

For CCK‐8 assay, 5 × 10^3^ NSCLC cells were seeded into 96‐well plates. At the indicated time, 10μl CCK‐8 reagent (Beyotime) was added into each well for 2.5h at 37°C, followed by analysis of absorbance at 450nm with automatic microplate reader. Cell cycle was performed using PI staining (BD Biosciences). Subsequently, the percentage of cells at each phase (G0/G1, S, and G2/M) was analyzed by ModFit LT5.0 software.

### 2.6 Luciferase reporter assay

The full‐length sequences of circ‐CMPK1 and *cyclin D1* 3′‐UTR with wild‐type or mutated miR‐302e binding site were synthesized and cloned into psi‐CHECK2 (Promega) vector to construct luciferase reporter vectors. After that, miR‐302e or control mimics were co‐transfected with above vectors into A549 and H460 cells by Lipofectamine 2000 (Invitrogen), respectively. After 48 hr, cells were collected and the relative luciferase activities were measured using the Dual Luciferase kit (#E2920, Promega) and calculated by the ratio of the intensity of the firefly luciferin to renilla fluorescein.

### 2.7 Biotin‐labeled RNA pull‐down assay

The wild‐type or mutated miR‐302e biotin‐labeled probe (RiboBio) was transfected into A549 and H460 cell lines by Lipofectamine 2000 (Invitrogen). 48 hr after transfection, cells were washed and the lysates were collected, followed by incubation with streptavidin‐coupled magnetic dynabead (Invitrogen) for 2h at 37°C. Finally, miR‐302e‐bound circ‐CMPK1 was eluted and its expression level was detected by qRT‐PCR.

### 2.8 Western blot

The indicated vectors were respectively transfected into A549 and H460 cells by Lipofectamine 2000 (Invitrogen). After 48 hr, the proteins in each group were extracted using RIPA lysis buffer added with protease inhibitors. Then, quantification of protein was performed with Pierce BCA Protein Assay Kit (Invitrogen), followed by transfer, blocking, incubation with anti‐*cyclin D1* (#ab226977, Abcam) primary antibody and HRP‐conjugated secondary antibody. The blot was visualized with LumiBlue™ ECL solution (Expedeon).

### 2.9 In vivo animal experiment

To establish xenograft tumor model, 5 × 10^6^ A549 cells transfected with the indicated oligonucleotides or vectors were injected subcutaneously into the armpit of BALB/c nude mice (*n* = 5 in each group). All nude mice were male with 4–6‐week‐old and purchased from the Chinese Academy of Science. The volume of the tumor was measured every 7 days. 35 days after the injection, nude mice were all sacrificed and the tumor tissues were obtained and weighed. All procedures in the animal experiment were approved by the Animal Care Committee HeNan Provincial Chest Hospital (NO. HNPCH1523).

### 2.10 Statistical analysis

The Student's *t* or Chi‐square test was used for comparison between two groups. The survival curves of NSCLC patients with low and high miR‐302e or circ‐CMPK1 were determined by Kaplan–Meier plot and calculated by log‐rank test. Pearson's correlation coefficient was employed to assess the correlation between circ‐CMPK1 and miR‐302e or *cyclin D1* expression in NSCLC tissues. The statistical results and figures in this study are automatically generated by Graph‐pad prism 7.0 software. All experiments were at least three effective biological replicates. * *p* < .05, ** *p* < .01, *** *p* < .001.

## 3 RESULTS

### 3.1 miR‐302e is significantly decreased in NSCLC cell lines and tissues

**Figure 1:**
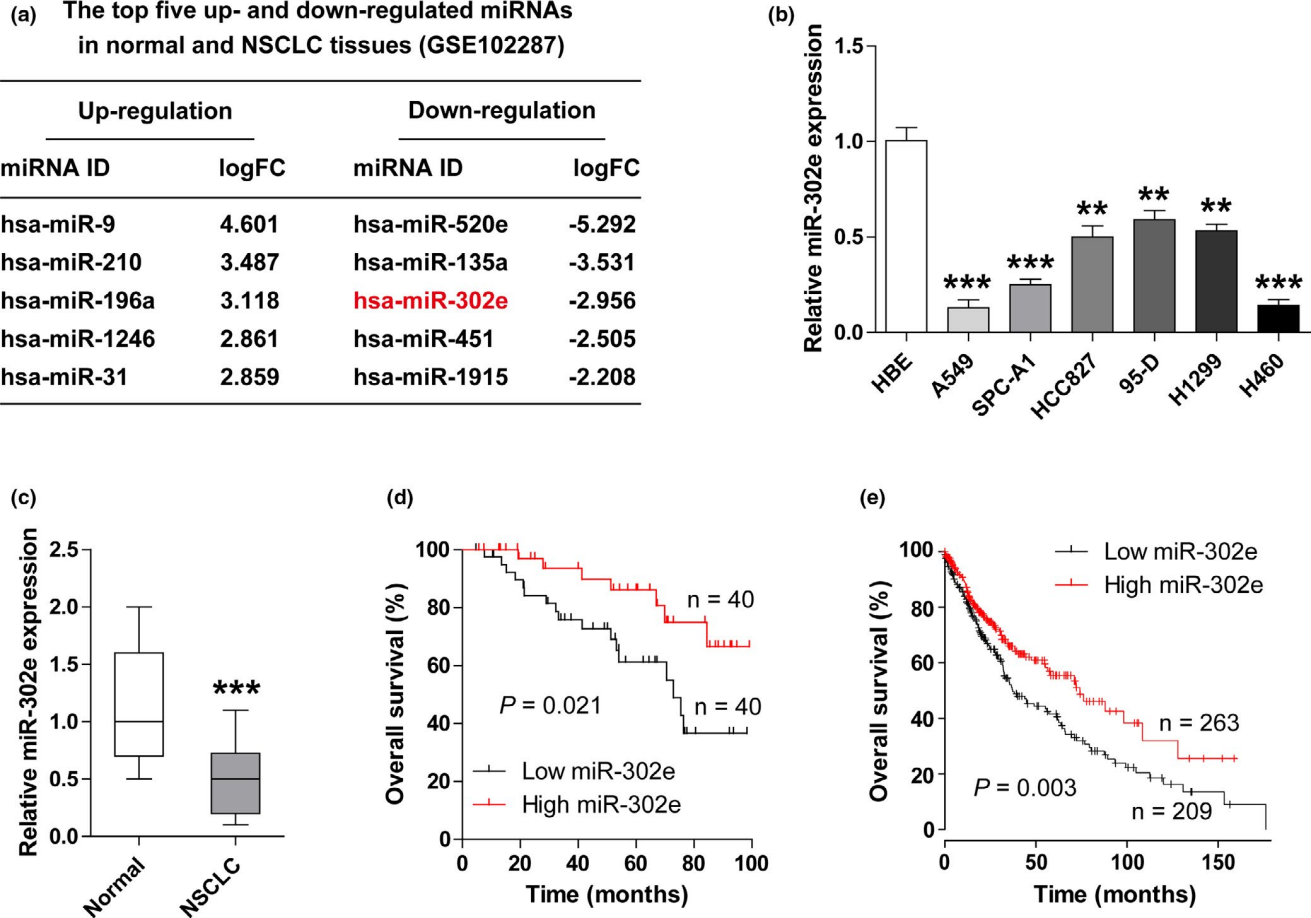
miR‐302e is frequently down‐regulated in NSCLC cells and tissues. (a) The top five up‐ and down‐regulated miRNAs in normal and NSCLC tissues (http://www.ncbi.nlm.nih.gov/geo/query/acc.cgi?acc=GSE102287). FC, Fold change. (b, c) qRT‐PCR analysis for miR‐302e expression in NSCLC cell lines (b) and tissues (c). (d, e) The survival curves of NSCLC patients with low and high miR‐302e expression in our (d) or KM‐plotter (e) survival data (http://kmplot.com/analysis/). ** *p* < .01, *** *p* < .001

To identify NSCLC‐related miRNAs, we first analyzed the GEO database (http://www.ncbi.nlm.nih.gov/geo/query/acc.cgi?acc=GSE102287) using the GEO2R method (https://www.ncbi.nlm.nih.gov/geo/geo2r/?acc=GSE102287). The top five up‐regulated (miR‐9, miR‐210, miR‐196a, miR‐1246, and miR‐31) and down‐regulated (miR‐520e, miR‐135a, miR‐302e, miR‐451, and miR‐1915) miRNAs in NSCLC are shown in Figure 1a. Due to the roles of other dysregulated miRNAs in NSCLC have been reported in addition to miR‐302e and miR‐1915, we chose miR‐302e and miR‐1915 for investigation. The preliminary experiment results showed that there was no significant difference in miR‐1915 expression between NSCLC and adjacent normal tissues (data not shown). However, the endogenous expression of miR‐302e was dramatically decreased in NSCLC cells and tissues (Figure 1b,c), which was consistent with the result of http://www.ncbi.nlm.nih.gov/geo/query/acc.cgi?acc=GSE102287. Moreover, decreased miR‐302e was closely associated with larger tumor size, lymph node metastasis, advanced clinical stage, and poor differentiation (Table 1). More importantly, NSCLC patients with low miR‐302e expression had shorter survival time than that with high miR‐302e expression (Figure 1d), and this result was also confirmed by the survival curves from KM‐plotter database with large samples (http://kmplot.com/analysis/) (Figure 1e). In all, these findings indicate that miR‐302e may play an important role in the pathogenesis of NSCLC.

### 3.2 Manipulation of miR‐302e expression affects the proliferation of NSCLC cells

**Figure 2:**
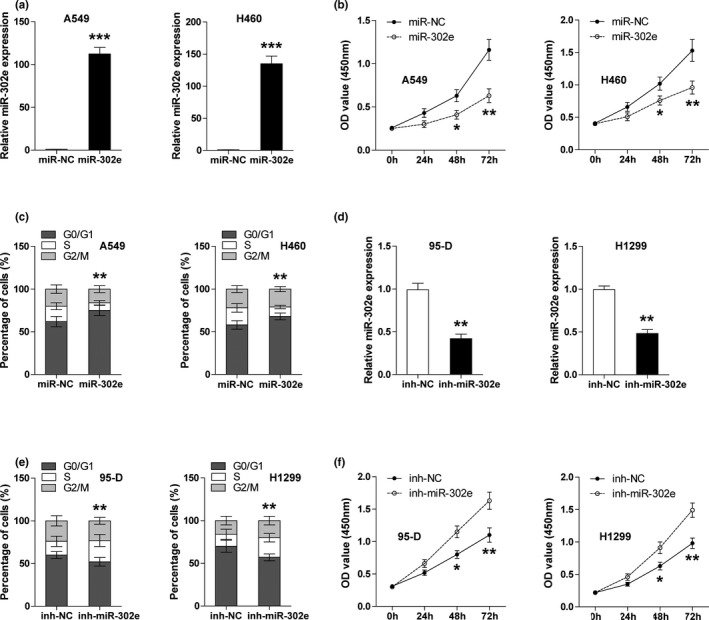
miR‐302e is a proliferation inhibiting factor in NSCLC cells. (a) qRT‐PCR analysis for miR‐302e expression in A549 and H460 cells transfected with control or miR‐302e mimics. (b) CCK‐8 assay at the indicated time in control or miR‐302e‐overexpressing A549 and H460 cells. The absorbance was recorded at 450nM. (c) Cell cycle analysis with PI staining in A549 and H460 cells. (d) qRT‐PCR analysis for miR‐302e expression in 95‐D and H1299 cells transfected with control or miR‐302e inhibitors. (e, f) CCK‐8 (e) and cell cycle (f) assays were used to test the proliferative abilities of control or miR‐302e‐silencing 95‐D and H1299 cells. * *p* < .05, ** *p* < .01, *** *p* < .001

Next, we respectively transfected control and miR‐302e mimics into A549 and H460 cells to evaluate the effects of miR‐302e overexpression on cell proliferation. The efficiency of miR‐302e overexpression was verified by qRT‐PCR (Figure 2a). As shown in Figure 2b, ectopic expression of miR‐302e weakened the ability of cells to proliferate, as determined by CCK‐8 assay. Likewise, more miR‐302e‐overexpressing A549 and H460 cells were arrested in G0/G1 phase as compared with the control cells (Figure 2c). To test whether knockdown of miR‐302e displays an opposite biological function, we chose 95‐D and H1299 cells to respectively transfect control and miR‐302e inhibitors because of their relatively high expression level of miR‐302e (Figure 2d). As expected, depletion of miR‐302e enhanced the proliferative capacity of 95‐D and H1299 cells, as determined by CCK‐8 and cell cycle assays (Figure 2e,f). Taken together, these data suggest that miR‐302e is a tumor growth inhibitor.

### 3.3 Circ‐CMPK1 acts as a sponge of miR‐302e in NSCLC cells

**Figure 3:**
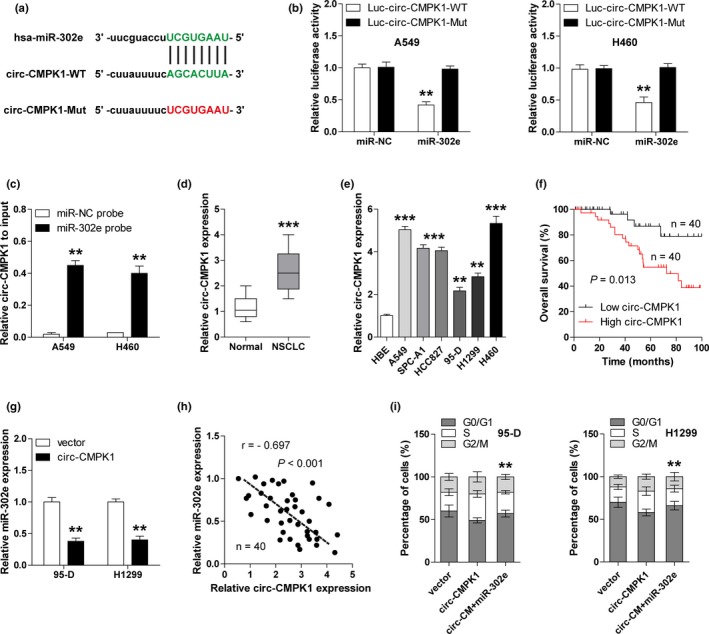
Circ‐CMPK1 acts as a sponge of miR‐302e in NSCLC cells. (a) The wild‐type and mutant sequence of circ‐CMPK1 and miR‐302e binding site. (b) Luciferase reporter assay for wild‐type and mutant circ‐CMPK1 luciferase vectors in A549 and H460 cells transfected with control or miR‐302e mimics. (c) RNA pull‐down assay was carried out in A549 and H460 cells transfected with biotinylated control or miR‐302e probe, followed by qRT‐PCR analysis for circ‐CMPK1 expression. (d, e) qRT‐PCR analysis for circ‐CMPK1 expression in NSCLC tissues (d) and cell lines (e). (f) The survival curves of NSCLC patients with low and high circ‐CMPK1 expression. (g) qRT‐PCR analysis for miR‐302e expression in 95‐D and H1299 cells with or without circ‐CMPK1 overexpression. (h) The correlation between circ‐CMPK1 and miR‐302e expression in NSCLC tissues. (i) Cell cycle analysis with PI staining in circ‐CMPK1‐overexpressing 95‐D and H1299 cells with or without miR‐302e overexpression. ** *p* < .01, *** *p* < .001

Numerous studies have shown that the expression levels of miRNAs are tightly controlled by circRNAs. To search for miR‐302e‐associated circRNAs, we analyzed the starBase V2.0 online prediction software (http://starbase.sysu.edu.cn) with the parameter of very high stringency (≥5). The results show that circ‐CMPK1 (hsa_circ_0012384) is the most likely circRNA to sponge miR‐302e owing to its highest clipReadNum value (6,002) (data not shown). And circ‐CMPK1 was mainly located in the cytoplasm, supporting its role as “miRNA sponge” (Figure S1). Next, the luciferase vectors with wild‐type or mutant miR‐302e binding site were constructed (Figure 3a). As shown in Figure 3b, miR‐302e overexpression significantly reduced the luciferase activity of wild‐type vector, but had no effect on the mutant one. To assess whether circ‐CMPK1 and miR‐302e are directly bound, we performed the RNA pull‐down assay with biotinylated miR‐302e and control probes. The results showed that more circ‐CMPK1 was enriched by miR‐302e probe compared with control probe in both A549 and H460 cells (Figure 3c). Importantly, we found that circ‐CMPK1 was highly expressed in NSCLC tissues and cells (Figure 3d,e), and closely related to poor prognosis (Figure 3f). Enforced expression of circ‐CMPK1 dramatically decreased the expression level of miR‐302e in 95‐D and H1299 cells (Figure S2, Figure 3g). And there was a strong negative correlation between circ‐CMPK1 and miR‐302e expression in NSCLC tissues (Figure 3h). Moreover overexpression of circ‐CMPK1 notably increased cell cycle acceleration in 95‐D and H1299 cells, however, this increase was abolished by simultaneously overexpression of miR‐302e (Figure 3i). Collectively, these results reveal that circ‐CMPK1 is a tumor growth promoting factor that can directly bind and inhibit miR‐302e expression.

### 3.4 Identification of circ‐CMPK1/miR‐302e/cyclin D1 ceRNA regulatory network in vitro and in vivo

**Figure 4:**
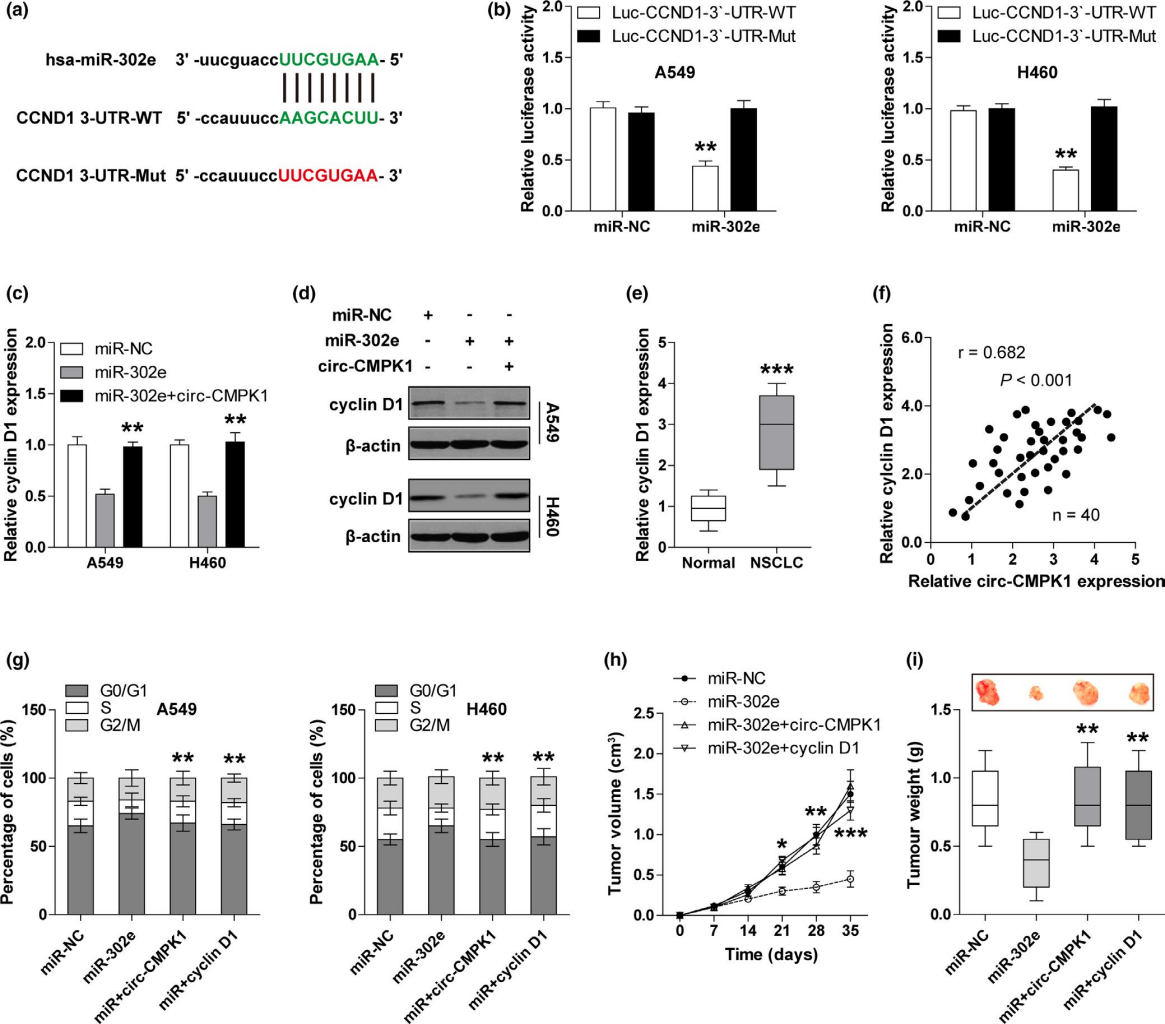
Novel circ‐CMPK1/miR‐302e/*cyclin D1* ceRNA regulatory network functions in vivo and in vitro. (a) The wild‐type and mutant sequence of miR‐302e and *cyclin D1* binding site. (b) Luciferase reporter assay for wild‐type and mutant *cyclin D1* 3′‐UTR luciferase vectors in A549 and H460 cells transfected with control or miR‐302e mimics. (c, d) qRT‐PCR (c) and western blot (d) assays for *cyclin D1* expression in miR‐302e‐overexpressing A549 and H460 cells with or without circ‐CMPK1 overexpression. (e) qRT‐PCR analysis for *cyclin D1* expression in adjacent normal and NSCLC tissues. (f) The correlation between circ‐CMPK1 and *cyclin D1* expression in NSCLC tissues. (g) Cell cycle assays with PI staining in miR‐302e‐overexpressing A549 and H460 cells with or without circ‐CMPK1 or *cyclin D1* overexpression. (h, i) The volume and weight of tumors in nude mice bearing miR‐302e‐overexpressing A549 cells with or without circ‐CMPK1 or *cyclin D1* overexpression (*n* = 5 in each group). * *p* < .05, ** *p* < .01, *** *p* < .001

Subsequently, we used the miRanda database (http://www.microrna.org/) to identify the downstream target of miR‐302e. The predicted result shows that there is a miR‐302e binding site in the 3′‐UTR of *cyclin D1* (CCND1) (Figure 4a). Also, overexpression of miR‐302e dramatically decreased the luciferase activity of wild‐type vector, but not the mutant one, implying that *cyclin D1* is the target of miR‐302e (Figure 4b). Furthermore, enforced expression of miR‐302e down‐regulated both mRNA and protein expression of *cyclin D1* in A549 and H460 cells, but this down‐regulation could be blocked by circ‐CMPK1 overexpression (Figure 4c,d). Importantly, *cyclin D1* expression was significantly increased in NSCLC tissues (Figure 4e) and strongly positively correlated with circ‐CMPK1 expression (Figure 4f). Functionally, the cell cycle arrest caused by miR‐302e overexpression was obviously reversed by circ‐CMPK1 or *cyclin D1* overexpression in A549 and H460 cells (Figure 4g). In an attempt to detect whether this circ‐CMPK1/miR‐302e/*cyclin D1* signal axis is also valid in vivo, we established the xenograft model by subcutaneously injecting A549 cells into nude mice (*n* = 5 for each group). The results showed the average volume and weight of tumors in nude mice bearing miR‐302e‐overexpressing cells were significantly smaller than those in the control mice (Figure 4h,i). Analogously, the inhibitory effect of miR‐302e on tumor growth was effectively counteracted by restoration of circ‐CMPK1 or *cyclin D1* expression (Figure 4h,i). Altogether, these above data suggest that circ‐CMPK1 functions as an oncogene in NSCLC by the regulation of miR‐302e/*cyclin D1* axis.

## 4 DISCUSSION

It is well known that miRNA plays a pivotal role in the regulation of cancer progression. To date, despite a relatively large proportion of miRNAs have been well functionally described, many members of this class have not yet been characterized in NSCLC. In this study, we found that miR‐302e, an endogenous miRNA that has not been previously explored in NSCLC, was significantly down‐regulated in NSCLC cell lines and tissues, which was closely related to malignant features and poor prognosis of patients with NSCLC. Functionally, ectopic expression of miR‐302e attenuated, while silencing of miR‐302e strengthened, the proliferative capacity of NSCLC cells. Stepwise investigations implied that miR‐302e acted as an intermediate bridge between circ‐CMPK1 and *cyclin D1*, that is, circ‐CMPK1 could elevate the expression of *cyclin D1* to promote NSCLC cells proliferation through weakening the inhibitory effect of miR‐302e on *cyclin D1* via directly binding with miR‐302e both in vitro and in vivo. Taken together, our data elucidate the potential mechanism of action of this novel circ‐CMPK1/miR‐302e/*cyclin D1* ceRNA regulatory axis in NSCLC, which promotes understanding of the aggressive progression of NSCLC.

Accumulating evidence shows that miRNA is involved in various cellular physiologic processes, including cell proliferation, apoptosis, migration, differentiation, and senescence (Chipman & Pasquinelli, 2019). Here we found that decreased miR‐302e was linked to accelerated cell proliferation. miR‐302e is a member of the miR‐302 cluster family, which also contains miR‐302a, miR‐302b, miR‐302c, and miR‐302d (Guo et al., 2017). Except for miR‐302e, other members in this family have been extensively studied and considered as tumor suppressors in many human cancers (Chen, Chen, & Ying, 2019; Li, Huo, Pan, Wang, & Ma, 2018). In our data, miR‐302e was observed to be significantly down‐regulated in NSCLC and identified as a tumor growth inhibitor. Further investigations are warranted to clarify whether miR‐302e also acts as a tumor suppressor in other tumors. Substantial studies have shown that miRNA regulates gene expression via promoting the degradation or inhibiting translation of its downstream target genes via pairing with their 3′‐UTR or CDS sequences (Adams, Parsons, Walker, Zhang, & Slack, 2017). Herein, we found that miR‐302e could bind to the 3′‐UTR of *cyclin D1* to inhibit its expression. *cyclin D1*, a well‐known proliferation‐promoting factor, can drive G1 to S phase progression by forming the active complexes with cyclin‐dependent kinases 4 (CDK4) or CDK6 (Musgrove, Caldon, Barraclough, Stone, & Sutherland, 2011). Thus, these results indicate that miR‐302e exerts the anti‐proliferation effect at least in part by inhibiting *cyclin D1* activity in NSCLC.

CircRNA is a recent research hotspot in the field of non‐coding RNA, which has a covalently closed loop structure and participates in the occurrence and development of human cancers by acting as an oncogene or a tumor suppressor mainly through adsorbing and suppressing miRNAs, that is, ceRNA mechanism (Shang, Yang, Jia, & Ge, 2019; Verduci, Strano, Yarden, & Blandino, 2019). In this study, we found that circ‐CMPK1 (hsa_circ_0012384) was frequently overexpressed in NSCLC cell lines and tissues. Subsequent mechanism experiments showed that increased circ‐CMPK1 could directly interact with miR‐302e and functioned as a ceRNA to elevate oncogenic *cyclin D1* expression by inhibiting miR‐302e activity, thereby promoting the progression of NSCLC. Importantly, the miR‐302e‐induced tumor suppressive effect could be effectively reversed by circ‐CMPK1 or *cyclin D1* overexpression both in vitro and in vivo, implying that the ceRNA regulatory network of circ‐CMPK1/miR‐302e/*cyclin D1* is objectively present and plays an important role in NSCLC. Therefore, we describe a novel oncogenic circRNA that can facilitate the growth of NSCLC by regulating the miR‐302e/*cyclin D1* axis, which enriches our understanding of circRNA in NSCLC. Whether circ‐CMPK1 is involved in the progression of other malignancies is highly worthy of further study.

Of note, recent studies have shown that non‐coding RNA, including miRNA and circRNA, can be used as a feasible and effective prognostic biomarker in human cancers (Beermann, Piccoli, Viereck, & Thum, 2016). For example, miR‐424‐5p/circ‐LARP4 (Zhang et al., 2017), miR‐126/circ‐ANKS1B (Zeng, et al., 2018; Zhang et al., 2013), miR‐192‐5p/circ‐SMARCA5 (Gu et al., 2018; Yu et al., 2018), and miR‐106b/circ‐MYLK (Lee et al., 2018; Zhong et al., 2017) were identified as the promising prognostic biomarkers of gastric cancer, breast cancer, hepatocellular carcinoma, and bladder cancer, respectively. Likewise, our clinical data showed that NSCLC patients with low miR‐302e or high circ‐CMPK1 expression had shorter survival time than those with high miR‐302e or low circ‐CMPK1 expression, revealing that miR‐302e or circ‐CMPK1 may be a potential prognostic predictor for patients with NSCLC.

Collectively, in this study, we for the first time uncover the important regulatory role of circ‐CMPK1/miR‐302e/*cyclin D1* ceRNA network in NSCLC, which provides promising prognostic indicators and potential therapeutic targets for NSCLC patients.

## CONFLICT OF INTEREST

The authors have no competing financial interests to be declared.
