# Supplementary material for: Circular RNA circ‐CMPK1 contributes to cell proliferation of non‐small cell lung cancer by elevating cyclin D1 via sponging miR‐302e
Source: Mol Genet Genomic Med. 2019 Dec 21;8(2):e999. doi: 10.1002/mgg3.999 (PMC7005605; doi:10.1002/mgg3.999)
Supplement: Supplementary file 1 [file MGG3-8-e999-s001.doc]

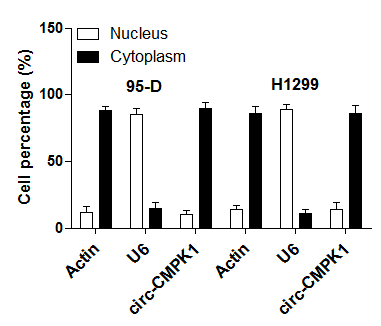


Supplementary Figure 1. qRT-PCR analysis of the subcellular location of circ-CMPK1 in NSCLC cells. Actin and U6 were selected for the control references of cytoplasmic and nuclear fractions, respectively.


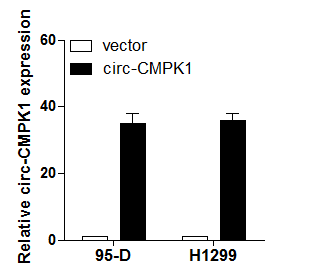


Supplementary Figure 2. qRT-PCR analysis of circ-CMPK1 expression in 95-D and H1299 cells transfected with control or circ-CMPK1 expression vector.
